# The relationship between reduced child spacing and depression in mothers: A cross-sectional study in Karachi, Pakistan

**DOI:** 10.1371/journal.pgph.0007190

**Published:** 2026-09-10

**Authors:** Syed Abbas Moazzam Kazmi, Muhammad Mushahid Hussain Rizvi, Sameen Nasir, Shahnoor Ahmed, Hawra Hussain, Haleema Sadia, Ayesha Siddiq, Aleena Asif, Maham Jaskani, Zulfiqar Jogezai, Noor us Sabahat, Abdul Basit Afzal, Asaad Ahmed Nafees

**Affiliations:** 1 Medical College, The Aga Khan University, Karachi, Pakistan; 2 Department of Community Health Sciences, The Aga Khan University, Karachi, Pakistan; Centre of Biomedical Ethics and Culture, PAKISTAN

## Abstract

Short inter-birth intervals may increase maternal psychological stress and have been hypothesized to contribute to depressive symptoms. We assessed the association between recent birth spacing and depression in mothers in Karachi, Pakistan. We conducted a cross-sectional, interviewer-administered survey of mothers recruited via convenience sampling from public parks and shopping malls in Karachi. Eligible participants were mothers with ≥2 children who had delivered within the past five years; women who were currently pregnant or <2 months postpartum were excluded. Depressive symptoms were measured using the Patient Health Questionnaire-9 (PHQ-9); the primary outcome was PHQ-9 ≥ 10. Birth spacing was calculated as the interval between the two most recent live births and categorized as <24 months, 24–32 months, and ≥33 months (reference). We used logistic regression to estimate crude and adjusted odds ratios (aOR), adjusting for maternal age, education (formal vs none), number of children (2 vs ≥ 3), and household income (<50,000 vs ≥ 50,000 PKR). Of 202 completed questionnaires, 15 were excluded due to missing key exposure data, leaving 187 mothers for analysis; adjusted models included 181 participants. Overall, 37/187 (19.8%) screened positive for probable depression (PHQ-9 ≥ 10). Compared with spacing ≥33 months, the adjusted odds of PHQ-9 ≥ 10 were aOR 2.39, 95% CI 0.84–6.75 for spacing 24–32 months, and aOR 2.17, 95% CI 0.79–5.96 for <24 months. No formal education was associated with higher odds of PHQ-9 ≥ 10 (aOR 4.62, 95% CI 1.50–14.20). In this sample of mothers in Karachi, shorter birth spacing was not significantly associated with higher odds of probable depression. Larger, probability-based studies are needed to clarify the relationship between birth spacing and depression.

## Background

Depression affects over 120 million people worldwide and affects women twice as much as men. Despite treatment plans, depressive disorders continue to impose significant burdens on patients. Certain symptoms, such as cognitive impairment or social dysfunction may decrease an individual’s quality of life. Depression hampers productivity and causes patients to struggle to maintain healthy social relationships with friends and family, leading to poor social dynamics [1]. Depression is prevalent in mothers, especially in the postpartum period, which lasts a few months after birth. A meta-analysis study reported that postpartum depression (PPD) is found in approximately 12% of women. However, depression is common among mothers even after this period, which accounted for an extra 5% of women [2]. A study conducted in Ghana found that depression was prevalent in 17% of mothers who had children less than 5 years of age [3].

Maternal depressive symptoms may persist or recur beyond the conventionally defined postpartum period, and their determinants extend beyond pregnancy-related biological changes to include sustained caregiving demands, socioeconomic disadvantage, relationship difficulties, and limited social support. Accordingly, depression among mothers of young children should not be considered exclusively a postpartum phenomenon [4].

Various determinants affect the average interval between births among women, including age at marriage, education, and socioeconomic status [5]. The major risk factors for reduced child spacing (CS) include women’s lack of understanding about its adverse effects on their own health, as well as their children’s health, lower access to and use of family planning and contraception services, and poor access to healthcare facilities, including individual, household, and community-based services [6,7].

Conversely, the major risk factors for maternal depression are a history of depression and increasing maternal age. Additionally, comorbidities may increase the risk for maternal depression; As an example, diabetes increases the risk for PDD by a factor of 1.5 [8]. It is also well-established that less family support is strongly linked to PPD [9]. In South Asia, the risk factors for maternal depression include a low socioeconomic status, disappointment with the sex of the child, and a dysfunctional relationship with a mother-in-law or partner [10].

Recent evidence from Pakistan similarly emphasizes that depression among married women is socially patterned. Analyses of married women of reproductive age have identified socioeconomic disadvantage, restricted autonomy, adverse marital circumstances, and limited support as relevant determinants of depressive symptoms [11–13]. Studies from Pakistan have also documented strong associations between intimate-partner violence and depression, anxiety, and impaired quality of life [14]. Marriage should therefore not be assumed to be uniformly protective: its mental-health implications depend on relationship quality and family structure.

Although a study found that symptoms of prenatal depression are associated with an increase in the odds of shorter interpregnancy intervals [15], there is insufficient evidence on short interpregnancy intervals leading to depression [16]. Existing longitudinal evidence has more clearly demonstrated the reverse pathway: depressive symptoms before or during pregnancy may be followed by shorter subsequent interpregnancy intervals [15]. This may be because depression may influence contraceptive use, pregnancy intention, relationship dynamics, and engagement with healthcare, while closely spaced births may independently increase cumulative caregiving and socioeconomic strain. However, we believe that since the varied birth spacing is linked to maternal physical health, it may also affect mental health. Therefore, the reduced CS could be linked to depression in mothers.

Pakistan’s fertility rate in 2020 was 3.4. In comparison, the fertility rates in India, Bangladesh, and the United States were 2.2, 2.0, and 1.6, respectively [17]. Although Pakistan’s median birth interval is 28.2 months, according to the Pakistan Demographic and Health Survey (PDHS) 2017–2018, 37% of the births occur within 24 months of the preceding birth [7], with the worldwide median birth interval being 32.1 months, indicating that, on average, birth intervals are shorter in Pakistan compared with the rest of the world [18]. A study across 21 lower-middle-income countries (LMICs) revealed that Pakistan has one of the highest percentages (60%) of short birth-to-pregnancy intervals (<23 months after birth) with a 31% unmet need for spacing and 29% unmet need for limiting [7].

The high prevalence of short intervals in Pakistan therefore occurs alongside persistent unmet need for contraception and marked socioeconomic inequalities in reproductive autonomy. These conditions may also overlap with determinants of depression, creating potential confounding by education, household resources, marital relationships, and access to healthcare [13,19].

In Pakistan, according to a study which utilized The Center for Epidemiology Studies Depression (CES-D) scale, 65% of married women in Karachi were depressed [20]. Regarding PPD, estimates suggest that its prevalence in Pakistan may range from 28% to 63%, which would place the country amongst the highest in Asia [21]. For comparison, worldwide estimates of PPD prevalence range from 6.5% to 20%, indicating that Pakistan has a significantly greater burden of PPD than the global average [22]. Due to a lack of awareness of maternal depression (especially PPD) and other social factors, women in Pakistan who suffer from PPD do not actively seek out help for their condition, leading to potentially serious long-term complications [9]. Therefore, it is difficult to quantify its prevalence in the country definitively.

Our study determined whether a relationship exists between reduced child spacing (CS) in families and depression in mothers. We identified no studies in Pakistan exploring this specific relationship. While existing literature discusses birth spacing and its impact on a mother’s physical health [18], its effects on maternal mental health remain largely unaddressed, highlighting a gap in information. Decreased birth spacing can hinder the mother’s physical recovery, exacerbate nutrient deficiencies, and limit time for self-care due to the demands of childcare. This may affect her sleep, nutrition, and mood, and is often compounded by financial strain. These cumulative stressors can increase the risk of postpartum physical and mental health issues, including depression. The findings of our study may be utilized to help counsel mothers and their families about risk factors that may lead to depression, potentially benefiting maternal well-being, child development, and overall family dynamics.

We believe that during the years when children are highly dependent on their mother, the mother’s social and professional lives are affected, and they may develop mental illnesses, such as depression. We aimed to determine the relationship between reduced child spacing and depression in mothers in Karachi, Pakistan. Additionally, we determined the relationship between other risk factors and depression in mothers.

## Methodology

### Ethics statement

Approval by the Ethics Review Committee (form number 2022-8027-22867) of The Aga Khan University was acquired before the initiation of our data collection. The objectives of the study were clearly explained to respondents and written informed consent was obtained from voluntary participants before the questionnaire was administered. Participants were guaranteed confidentiality and anonymity, and the privacy of the collected data was ensured throughout the study. Furthermore, considering cultural and social norms and taboos pertaining to maternal health, depression, and contraceptive methods, all the questions in the questionnaire were asked in a respectful and non-intrusive manner.

### Study design and setting

We conducted a cross-sectional study in which we explored the relationship between reduced CS and depression in mothers. With a population of approximately 20 million [23] Karachi is one of the world’s largest cities. As a culturally and ethnically diverse city, Karachi is home to many different languages, with Urdu being the most commonly spoken [24].

Data collection took place from October 1st to 11th, 2022. We used non-probability convenience sampling because data collection was conducted as part of a time-limited Community Health Sciences rotation. Twelve third-year medical students collected data, so a pragmatic approach was required to recruit eligible participants within the available timeframe. Data collection occurred during standard working hours (9:00 AM–5:00 PM) in public parks, shopping malls, and in various neighborhoods (Clifton, Defence, Tariq Road, Bahadurabad, Garden), where team members went door to door. These particular locations represent various socioeconomic groups in the city. Permission to conduct recruitment (if necessary) was obtained from the relevant site authorities.

### Sample size calculation

Sample size was estimated a priori using OpenEpi’s sample size calculator [25] for an unmatched exposure-outcome comparison, assuming a two-sided α = 0.05, 80% power, and a 1:1 ratio of exposed to unexposed. Because there were no prior Pakistani studies quantifying the association between short birth spacing and depressive symptoms, the expected effect size was uncertain. However, a study which looked at depression in married women in Karachi had an OR of 3 [20]. We therefore planned for a moderate effect size (OR≈2.5) as a pragmatic planning assumption informed by the magnitude of associations reported for depression-related risk factors in similar populations in Karachi, while recognizing that the true effect, if present, could be smaller. Under these assumptions (with an assumed exposure prevalence among controls of ~40%), the minimum required sample size was 170, and we aimed to recruit slightly above this target to account for nonresponse.

### Eligibility and recruitment

Participants included women between the ages of 18–45 living in Karachi. Women over 45 were not included as they comprise the perimenopausal and/or menopausal age. Eligible participants were mothers with at least two living children who had delivered a child within the past five years, were able to provide informed consent, and were willing to complete an interviewer-administered questionnaire. We excluded women who were currently pregnant or who had delivered within the past two months, because we intended to focus on depressive symptoms outside the immediate peripartum period, and we did not measure postpartum depression specifically. Depressive symptoms were assessed at the time of the interview using the PHQ-9 and therefore reflect current symptoms at a single time point.

We focused on mothers with a birth in the past five years because early childhood is a period of high caregiving demand, and short birth intervals during this period may plausibly contribute to maternal psychological strain and reduced recovery time between pregnancies.

### Consent, data collection, and study tools

We conducted interviewer-administered questionnaires with willing female respondents. These interviews began with an introduction of the team and study, followed by a screening question asking how many children the respondent had. Once confirmed that the respondent had at least 2 children and was eligible for the study, participants were informed that their participation was entirely voluntary, that they could decline to answer any questions, and that they could withdraw from the study at any time without penalty. Privacy and confidentiality were assured, and no personal identifiers were included in the dataset. The consent process involved the participant reading or being read the consent form, followed by signing (or thumb-printing for illiterate participants) in the presence of the interviewer, who also signed as a witness. Our team collected data digitally, via a questionnaire on Google Forms, which was filled in by the interviewer based on the participants’ responses.

We collected data using a pre-validated questionnaire, the Patient Health Questionnaire-9 (PHQ-9). PHQ-9 is a study tool used to assess the severity of depression, and it can also be used to evaluate the response to any treatment that may be administered. During our study, we used English and Urdu versions of the PHQ-9, as previous studies have indicated that it is a valid and reliable instrument to screen, rate, and monitor depression in Pakistan [26,27].

The data collected was saved in an Excel sheet connected to the Google Form. Lastly, we ensured that all participants physically signed the consent form on a hard copy; the forms and data will be kept for seven years after the completion of the study, and will be discarded according to the institutional policy.

### Variables and operational definitions

The main independent variable was the duration of CS, which we calculated using the children’s dates of birth through participant recall. We categorized CS as <24 months, 24–32 months, and ≥33 months (reference), consistent with commonly used public health thresholds and World Health Organization (WHO) guidance, which recommends that the mother wait for at least 24 months after a live birth before attempting a subsequent pregnancy. Thus, an adequate birth interval (i.e., 24 + 9 months) should be 33 months or longer [28]. Such an interval reduces the risk of maternal and perinatal complications. We defined CS as the interval between two consecutive live births. We selected the most recent inter-birth interval a priori because it is the interval most proximal to the mother’s current caregiving demands and postpartum recovery, and therefore most relevant to current depressive symptoms assessed at the time of interview.

Other variables, such as current age, employment status, education level, marital status, number of children, age at first childbirth, unplanned child, history of miscarriage, pregnancy complications, history of depression, history of antidepressant use, monthly income, age at marriage, family structure, attitude toward children, attitude toward children’s gender, support of spouse, support of mother-in-law, and support of in-laws were also assessed via the questionnaire.

Attitudes were assessed using single-item questions asking whether the mother had any negative thoughts/attitudes toward (a) her children and (b) her child’s gender. Responses were recorded as yes/no and coded as negative attitude present versus no negative attitude for analysis. Perceived support from the spouse, mother-in-law, and in-laws was assessed using single-item Likert-scale questions (1 = strongly disagree, 2 = disagree, 3 = neutral, 4 = agree, and 5 = strongly agree). For analysis, responses were dichotomized into “support” vs “no support.” Likert responses 1–2 were coded as “no support” and responses 3–5 were coded as “support.” Because these were single-item measures (not multi-item scales), internal consistency reliability (Cronbach’s α) was not applicable.

The dependent variable in this study was depression in mothers, measured using PHQ-9. The primary outcome was probable depression defined as PHQ-9 ≥ 10, with sensitivity analyses also being conducted. Depression in mothers can be a result of a multitude of combinations of stressors, and these stressors may be biological, psychological, or social in nature, such as a lack of family support, or domestic violence. This study focused explicitly on one factor – reduced CS. Therefore, we did not take into account maternal depression, which is an umbrella term used for antepartum and postpartum depression [29]. In other words, depression due to stressors related to child rearing, for example, increased housework as a result of taking care of children, was taken into consideration. It is important to note that in this study, the terms “depression in mothers” and “depressive symptoms in mothers” are used interchangeably.

### Statistical analysis

All analyses were conducted using Stata (StataCorp. 2017. Stata Statistical Software: Release 15. College Station, TX: StataCorp LLC). The time interval between two consecutive births was considered during the study. In cases where a participant had more than 2 children, the spacing between the last and second last child was calculated. Quantitative variables such as maternal age, age at first childbirth, and age at marriage were analysed as continuous variables. Variables such as household income, education, marital status, and perceived social support were recategorized where appropriate.

The primary exposure was the inter-birth interval between the two most recent live births, categorized as <24 months, 24–32 months, and ≥33 months (reference). We estimated crude odds ratios (cOR) using univariable logistic regression and adjusted odds ratios (aOR) using multivariable logistic regression with a prespecified confounder set based on a conceptual causal framework (maternal age [continuous], education [formal vs no formal], number of children [2 vs ≥ 3], and household income [<50,000 vs ≥ 50,000 PKR]). The adjusted analysis used complete-case data with n = 181. Given the low level of missingness and the small number of observations excluded from multivariable models, multiple imputation was not performed.

The dependent variable in this study was depression in mothers. The primary outcome was probable depression, defined as PHQ-9 ≥ 10. Sensitivity analyses evaluated alternative outcome specifications: (i) PHQ-9 ≥ 5, (ii) ordinal PHQ-9 severity categories modeled using ordered logistic regression (no depression (0–4), mild (5–9), moderate (10–14), moderately severe/severe (≥15)) and (iii) continuous PHQ-9 score modeled using linear regression with robust standard errors. To account for the few responses, we combined moderately severe and severe depression.

We adjusted the primary model for maternal age, education, parity, and household income as upstream confounders. Variables plausibly on the causal pathway (e.g., psychosocial support measures) were not included in the primary model to avoid overadjustment. There were 37 participants with PHQ-9 ≥ 10; to reduce overfitting, we used a parsimonious prespecified adjustment set and limited the number of model parameters (EPV considered).

To assess model adequacy, we considered the events-per-parameter (EPV): there were 37 participants with PHQ-9 ≥ 10, and the primary adjusted model included 6 parameters (EPV ≈ 6.2). Multicollinearity was assessed using variance inflation factors (VIF). Model calibration and discrimination were evaluated using the Hosmer–Lemeshow goodness-of-fit test and the area under the ROC curve (AUC). Influence was assessed using leverage and standardized residual diagnostics, with a sensitivity analysis excluding high-leverage observations.

Missingness was summarized for variables included in Table 1 and the regression models. Missing data were minimal for analysis variables: education (1/187, 0.5%) and household income (5/187, 2.7%) had the highest missingness, while the exposure (birth spacing), PHQ-9 outcomes, age, and number of children were complete. The primary adjusted regression models were conducted using complete-case analysis, yielding an analytic sample of n = 181.

**Table 1:** Sociodemographic and obstetric characteristics of participants.

Variable	Frequency (n)	Percentage (%)
**Child spacing (n = 187)**		
≥ 33 months	102	54.5
24–32 months	38	20.3
< 24 months	47	25.1
**Current age (n = 187), mean±SD**	32.2 ± 5.6	
**Employment status (n = 187)**		
Employed	47	25.1
Unemployed	140	74.9
**Education level (n = 186)**		
Formal education	166	89.3
No formal education	20	10.8
**Marital status (n = 186)**		
Married	182	97.8
Unmarried	4	2.2
**Monthly income (n = 182)**		
≥ 50,000	90	49.5
< 50,000	92	50.5
**Age at marriage (n = 181), mean±SD**	22.2 ± 3.8	
**Family structure (n = 187)**		
Nuclear	63	33.7
Joint	118	63.1
Single-parent	6	3.2
**Number of children (n = 187)**		
2	94	50.3
≥ 3	93	49.7
**Age at first childbirth (n = 187), mean±SD**	23.7 ± 4.06	
**Unplanned child (n = 187)**		
Yes	62	33.2
No	125	66.8
**History of miscarriage (n = 187)**		
Prior miscarriage	61	32.6
No prior miscarriage	126	67.4
**History of pregnancy complications (n = 187)**		
Prior complications	36	19.3
No prior complications	151	80.7
**History of depression (n = 187)**		
Prior depression	15	8
No prior depression	172	92
**History of antidepressant use (n = 187)**		
Prior use	14	7.5
No prior use	173	92.5
**Attitude toward children (n = 186)**		
No negative attitude	161	86.6
Negative attitude	25	13.4
**Attitude toward children’s gender (n = 187)**		
No negative attitude	166	88.8
Negative attitude	21	11.2
**Support of spouse (n = 186)**		
Yes	147	79
No	39	21
**Support of mother-in-law (n = 187)**		
Yes	116	62
No	71	38
**Support of in-laws (n = 187)**		
Yes	104	55.6
No	83	44.4

The table summarizes the distribution of key demographic, socioeconomic, obstetric, and psychosocial variables among study participants. Data are presented as frequencies and percentages. Values are n (%) unless otherwise indicated. Denominators vary due to missing data; missingness was low overall. Exact denominators are shown.

## Results

### Study sample

**Fig 1:**
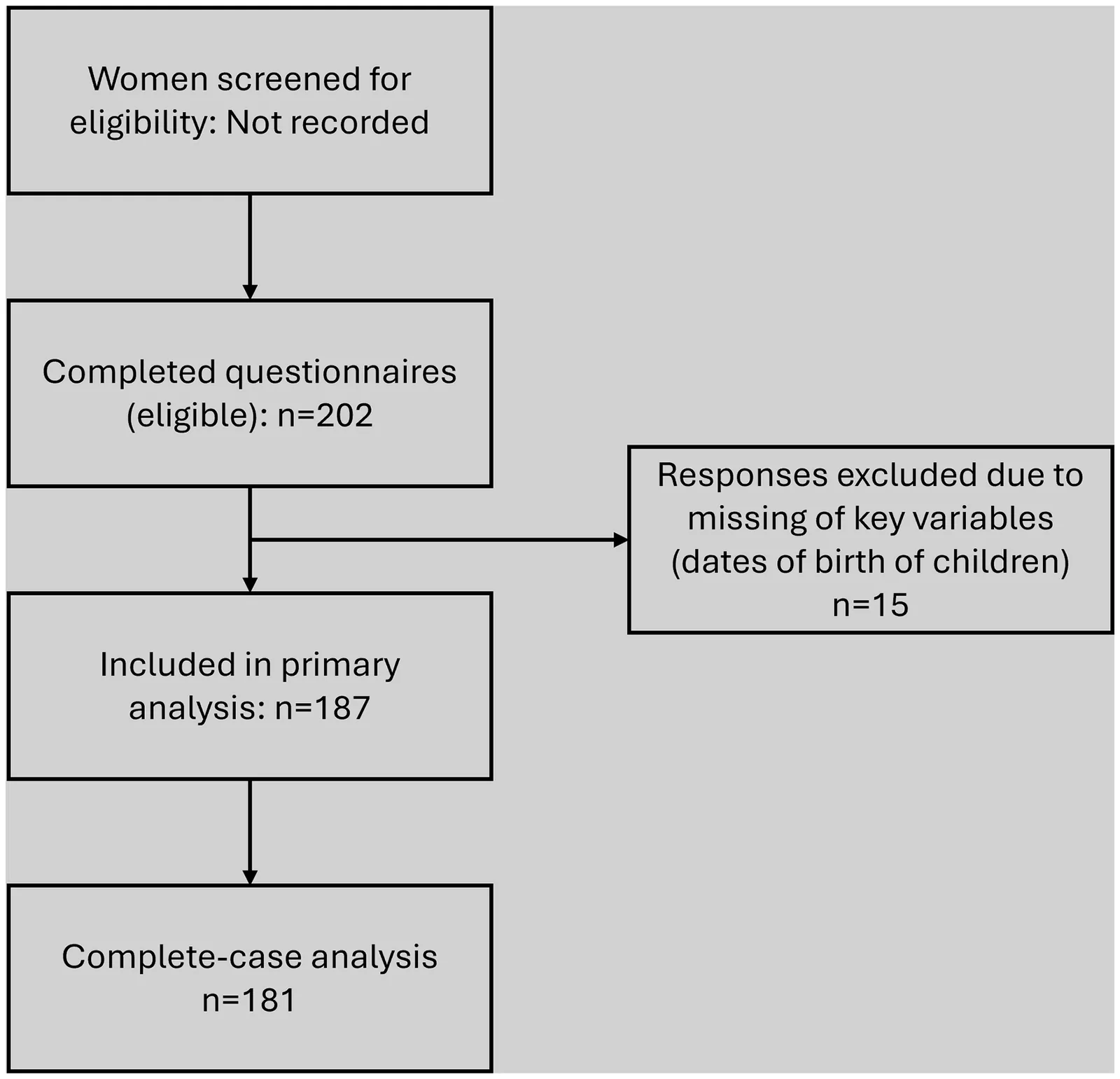
Participant flow through the study and analytic sample. The number of women approached and refusal rates were not recorded, but refusals appeared uncommon.

A total of 202 questionnaires were completed. For the present analysis, we excluded 15 responses with missing key exposure information required to calculate the birth interval (e.g., dates of birth), leaving 187 participants. Multivariable analyses were conducted as complete-case analyses (n = 181) due to minimal missing covariate data. Fig 1 summarizes the participant recruitment process.

### Participant characteristics and PHQ-9 distribution

**Fig 2:**
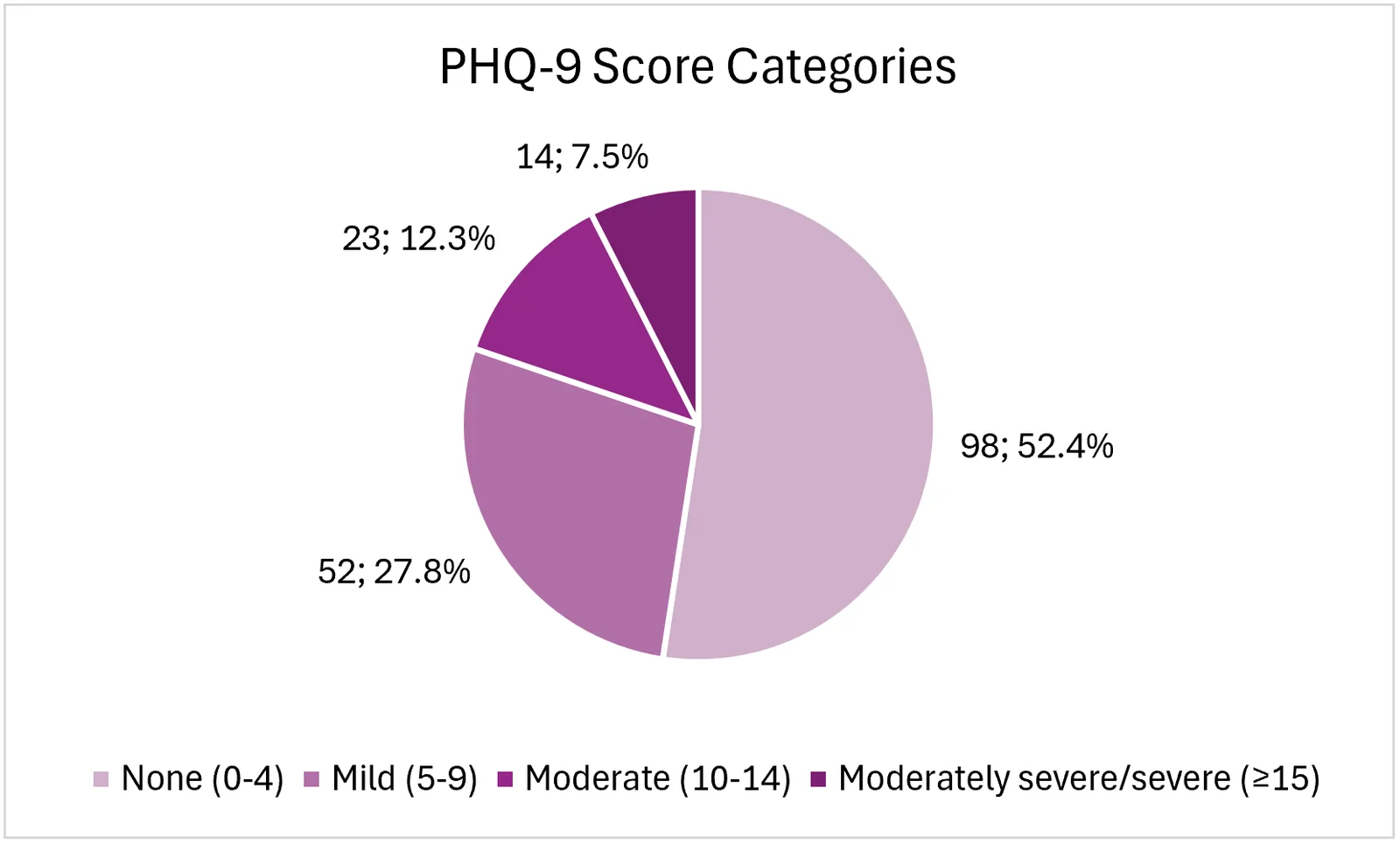
Distribution of depression severity in mothers based on PHQ-9 scores.

In our study, 89 participants (47.6%) had PHQ-9 scores ≥5, while 98 (52.4%) had scores below 5. This is summarized in Fig 2.

The qualitative variables were expressed as frequencies and percentages after data analysis. Among 187 participants, 37 (19.8%) screened positive for probable depression (PHQ-9 ≥ 10). The prevalence of PHQ-9 ≥ 10 was 10/47 (21.3%) among women with spacing <24 months, 10/38 (26.3%) among those with spacing 24–32 months, and 17/102 (16.7%) among those with spacing ≥33 months. This is summarised in Tables 1 and 2.

**Table 2:** Crude odds ratios (cOR) and adjusted odds ratios (aOR) between independent variables and depression.

Variable	n (%) with PHQ-9 ≥ 10	Crude OR (95% CI)	p-value	Adjusted OR* (95% CI)	p-value
**Child spacing (months)**					
≥ 33	17 (16.67)	Reference	–	Reference	–
24–32	10 (26.32)	1.35 (0.57–3.23)	0.50	2.39 (0.84–6.75)	0.10
< 24	10 (21.28)	1.79 (0.73–4.35)	0.20	2.17 (0.79–5.96)	0.14
**Maternal age (years)**	–	1.04 (0.98–1.12)	0.16	1.08 (1.00–1.17)	0.06
**Education level**					
Formal education	28 (16.87)	Reference	–	Reference	–
No formal education	9 (45.00)	4.03 (1.52–10.64)	0.01	4.62 (1.50–14.20)	0.01
**Household income (PKR)**					
≥ 50,000	16 (17.78)	Reference	–	Reference	–
< 50,000	21 (22.83)	1.37 (0.66–2.83)	0.40	0.83 (0.35–1.94)	0.66
**Number of children**					
2	13 (13.83)	Reference	–	Reference	–
≥ 3	24 (25.81)	2.17 (1.03–4.58)	0.04	1.51 (0.64–3.57)	0.34

Corresponding 95% confidence intervals (CI) and p-values are also given. Adjusted models were complete-case (n = 181) due to missing covariate data. *Adjusted for maternal age, education, parity/number of children, and household income (prespecified confounder set based on a conceptual causal framework). Reference category for spacing is ≥ 33 months.

### Primary regression analysis

In crude analyses (reference: ≥ 33 months), birth spacing was not significantly associated with PHQ-9 ≥ 10 (24–32 months: cOR 1.35, 95% CI 0.57–3.23; < 24 months: cOR 1.79, 95% CI 0.73–4.35).

After adjustment for maternal age, education, household income, and number of children, estimates were as follows: 24–32 months: aOR 2.39, 95% CI 0.84–6.75, p = 0.10; < 24 months: aOR 2.17, 95% CI 0.79–5.96, p = 0.14. The aOR for maternal age was 1.08 per year (95% CI 1.00–1.17; p = 0.06).

Compared with women with formal education, women with no formal education had significantly higher odds of PHQ-9 ≥ 10 (aOR 4.62, 95% CI 1.50–14.20, p = 0.01). This is shown in Table 2.

### Model diagnostics

The Hosmer–Lemeshow test yielded p = 0.752, and the area under the receiver operating characteristic curve was 0.689. The maximum VIF was 1.30. Effect estimates were similar after excluding observations classified as having high leverage.

### Sensitivity analyses

To assess robustness to outcome specification, we repeated the analysis using alternative PHQ-9 definitions while keeping the same exposure categorization and covariate adjustment set. Specifically, we modeled (i) PHQ-9 ≥ 5 using logistic regression, (ii) PHQ-9 severity categories using ordered logistic regression (reporting proportional odds ratios), and (iii) continuous PHQ-9 score using linear regression with robust standard errors. All sensitivity models adjusted for maternal age (continuous), education (formal vs no formal), number of children (2 vs ≥ 3), and household income (<50,000 vs ≥ 50,000 PKR), with ≥33 months as the reference spacing category. Note that ordered logistic regression estimates a proportional odds ratio under the proportional odds assumption. These results are shown in Table 3.

**Table 3:** Sensitivity analyses.

	PHQ-9 ≥ 5 (logistic)		PHQ-9 severity (ordered logit)*		PHQ-9 score (linear)	
Variable	Adjusted OR (95% CI)	p-value	Adjusted proportional OR (95% CI)	p-value	β (95% CI)	p-value
**Child spacing (months)**						
≥ 33	Reference	–	Reference	–	Reference	–
24–32	1.49 (0.65–3.37)	0.34	1.68 (0.79–3.58)	0.18	1.75 (−0.17 to 3.67)	0.07
< 24	1.56 (0.72–3.39)	0.26	1.69 (0.82–3.47)	0.15	0.93 (−0.91 to 2.78)	0.32
**Maternal age (years)**	1.00 (0.94–1.07)	0.89	1.02 (0.96–1.08)	0.48	0.08 (−0.06 to 0.22)	0.28
**Education level**						
Formal education	Reference	–	Reference	–	Reference	–
No formal education	3.12 (1.07–9.10)	0.04	3.38 (1.34–8.53)	0.01	3.29 (0.56 to 6.02)	0.02
**Household income (PKR)**						
≥ 50,000	Reference	–	Reference	–	Reference	–
< 50,000	0.79 (0.42–1.52)	0.49	0.78 (0.43–1.44)	0.43	−0.43 (−1.94 to 1.08)	0.58
**Number of children**						
2	Reference	–	Reference	–	Reference	–
≥ 3	1.01 (0.53–1.95)	0.97	1.20 (0.65–2.22)	0.56	0.66 (−0.94 to 2.27)	0.42

Adjusted odds ratios (aOR) for PHQ-9 ≥ 5, adjusted proportional odds ratios (aPOR) from ordered logistic regression for PHQ-9 severity, and adjusted mean differences (β) in PHQ-9 score with robust standard errors. All models adjust for maternal age, education, number of children, and household income; spacing reference category is ≥ 33 months. *None, mild, moderate, moderately severe/severe based on PHQ-9 cutoffs.

Sensitivity analyses were consistent with the primary findings. Using PHQ-9 ≥ 5 as the outcome, neither spacing <24 months (aOR 1.56, 95% CI 0.72–3.39, p = 0.260) nor spacing 24–32 months (aOR 1.49, 95% CI 0.65–3.37, p = 0.34) was significantly associated with depressive symptoms compared with spacing ≥33 months. When PHQ-9 was modeled as an ordinal severity outcome using ordered logistic regression, spacing <24 months (aPOR 1.69, 95% CI 0.82–3.47, p = 0.15) and 24–32 months (aPOR 1.68, 95% CI 0.79–3.58, p = 0.18) were similarly not significantly associated with greater symptom severity. In the continuous score analysis, spacing 24–32 months was associated with a higher mean PHQ-9 score compared with ≥33 months, but not statistically significant (β 1.75, 95% CI −0.17 to 3.67, p = 0.07); spacing <24 months showed a smaller and non-significant difference (β 0.93, 95% CI −0.91 to 2.78, p = 0.32).

Across sensitivity analyses, no formal education remained consistently associated with higher depressive symptom burden (PHQ-9 ≥ 5 aOR 3.12, p = 0.04; ordinal severity aPOR 3.38, p = 0.010; continuous PHQ-9 β 3.29, p = 0.02), whereas household income and number of children were not significantly associated after adjustment.

## Discussion

### Key findings

In this cross-sectional study of mothers in Karachi recruited from public spaces, we observed a high burden of both short CS and current depressive symptoms. Reduced birth spacing was common, and approximately one in five participants met criteria for probable depression using a PHQ-9 threshold of ≥10. Although estimates suggested higher odds of probable depression among mothers with shorter spacing (<24 months and 24–32 months) compared with spacing ≥33 months, the association was imprecise after adjustment and did not meet the prespecified threshold for statistical significance. In contrast, lack of formal education showed a consistent association with greater depressive symptom burden across multiple outcome specifications (PHQ-9 ≥ 10, PHQ-9 ≥ 5, ordinal severity, and continuous score). Together, these findings highlight that, in this sample, educational disadvantage appears to be a more robust correlate of current depressive symptoms than CS.

According to our study, the prevalence of reduced CS (<33 months) was 45.5%. The prevalence of depression (mild + moderate + moderately severe + severe) was found to be 47.6%, while a similar study in Karachi found it to be 65% [20]. Differences in sample characteristics, methodology, assessment tools, or contextual factors (such as socioeconomic status, access to healthcare, or cultural norms) may account for the variation in prevalence. These findings highlight a vulnerable population of mothers, many of whom face compounding reproductive, social, and economic challenges that may contribute to poor maternal health outcomes, including mental health issues. These contributing factors will be examined in further detail below.

### Child spacing and current depressive symptoms

Given the analyses using PHQ-9 ≥ 10 as the primary outcome and additional sensitivity models, the most defensible interpretation is that the association between spacing and current depressive symptoms was inconclusive in this sample. Point estimates were >1, but confidence intervals were wide and crossed the null. Limited precision is a plausible explanation; with 37 events (PHQ-9 ≥ 10) and a prespecified adjustment set, our multivariable models may not have been powered to detect modest associations. This is consistent with the fact that small samples can yield imprecise estimates even when true effects exist. Though shorter CS may increase depression risk due to cumulative biological stress, physical exhaustion, and psychological strain, a larger sample or more precise measures might yield significant results, indicating a need for further research.

Measurement limitations may also have attenuated associations. CS was derived from reported birth dates/intervals, so recall error could lead to exposure misclassification. Non-differential misclassification would generally bias effect estimates toward the null, potentially masking modest associations. Additionally, our exposure captures spacing between the two most recent births, which is conceptually aligned with current caregiving demands, but it may not fully capture cumulative reproductive history or earlier short intervals that could influence longer-term maternal mental health.

### Education and depressive symptoms

The most significant finding of our study was that women with no formal education were at higher odds of having probable depression (aOR = 4.62, 95% CI: 1.50-14.2, p = 0.01) compared to women without formal education. This significance was re-established in the sensitivity analyses (PHQ-9 ≥ 5: aOR = 3.12, 95% CI: 1.07–9.10, p = 0.04; PHQ-9 severity: aPOR = 3.38, 95% CI: 1.34–8.53, p = 0.01; continuous PHQ-9: β = 3.29, 95% CI: 0.56-6.02, p = 0.02). This finding is consistent with international literature. For instance, a large cohort study from Japan identified lower educational attainment as a significant risk factor for PPD [30], while a Chinese cross-sectional study found that primary-level education or less increased the risk of maternal depression (aOR = 1.47, 95% CI: 1.07-2.03) [31].

Although the cited studies focus on postpartum outcomes, the broader inference–that educational disadvantage is closely linked to maternal depressive symptoms–is supported by large-scale evidence. In the Japan Environment and Children’s Study (JECS), a cohort of over 90,000 mothers, lower educational attainment was associated with increased risk of PPD, enabling stable estimates and extensive covariate control [30]. Importantly, education likely operates through multiple pathways relevant to current depressive symptoms during childrearing: constrained economic opportunity, reduced autonomy in reproductive decision-making, barriers to accessing health information and services, and heightened exposure to household stressors. From a public health perspective, the consistency of the education association across our sensitivity analyses suggests that educational disadvantage may be a useful marker for targeted screening and linkage to support within community and primary care settings.

### Social, family, and gender norms

In the maternal mental health literature, particularly systematic reviews of PPD risk factors, lack of social support repeatedly emerges as a key correlate, alongside psychosocial adversity and household stress [32,33]. While our study measured perceived support through single-item questions (dichotomized from Likert responses), the conceptual importance of family support is especially salient in South Asian contexts where household decision-making and caregiving roles are often distributed across extended families. Evidence from Pakistan’s Bachpan cohort highlights the complexity of mother-in-law involvement: mother-in-law childcare can be protective in some contexts yet harmful in others, particularly under conditions of family conflict [34]. This aligns with the broader observation that extended families may provide instrumental support while also introducing relational stressors that affect mental wellbeing.

Furthermore, a meta-analysis reported higher PPD risk among mothers of female infants in countries like Nigeria, India, Turkey, and China, likely reflecting cultural preferences for male children. Data on Western countries are ambiguous [35]. Systematic reviews across settings suggest that the relationship between infant gender and maternal depression varies by context and may be stronger in settings with pronounced son preference. In Pakistan, these norms intersect with education, economic dependency, and household power dynamics, further supporting a determinants-based interpretation of maternal depressive symptoms rather than a narrow biomedical framing [36]. Overall, negative attitudes toward children and their gender can both result from and worsen depressive symptoms, forming a cycle that affects mother and child. Child gender may also interact with depression, influencing both maternal mental health and child development. Recognizing these patterns is key to designing targeted mental health interventions.

### Other sociodemographic correlates

In our adjusted models, household income was not independently associated with current depressive symptoms. Broader literature shows that socioeconomic status (SES) is related to depression [37,38], where low income is associated with higher odds for depression [39]. However, the strength and detectability of associations vary depending on how SES is measured (for example, education vs. income vs. employment) along with local context and residual confounding. Consistent with this, another study reported that employment, compared with unemployment, was independently associated with a lower prevalence of depressive symptoms during pregnancy. In that same study, household income and education were not associated with depressive symptoms after mutual adjustment for employment, income, and education, suggesting that employment-related dimensions of SES may be more salient than income alone in explaining depressive symptoms [40]. In our study, income was dichotomized and measured at one time point, which may have limited sensitivity to capture gradients in financial strain; moreover, education (a more stable marker of social position and agency) showed a more consistent association with depression than income. These findings suggest that in this setting, structural disadvantage captured by educational attainment may be more predictive of maternal mental health than household income alone, or that income effects may operate through pathways not fully captured by a binary income measure.

We observed no statistically significant independent association between having ≥3 children (vs 2) and current depressive symptoms after adjustment. Prior evidence on parity and depression is inconsistent and suggests that any association is likely context-dependent. For instance, a large prospective cohort study in mid-to-late life reported higher prevalence and incidence of depressive symptoms with increasing parity among multiparous women in high and upper-middle socioeconomic groups, whereas no such association was observed among women in low and lower-middle socioeconomic groups [41]. These findings imply that socioeconomic position may modify the relationship between parity and maternal mental health, potentially through differences in role strain, economic trade-offs, childcare demands, and social expectations. In our setting, extended-family living arrangements, informal childcare support, and cultural norms surrounding family size may reduce the independent psychological burden associated with higher parity.

Maternal age showed no significant association with current depressive symptoms in our adjusted model. Prior evidence indicates that age effects on maternal depression can be heterogeneous across settings and time windows, with some studies suggesting increased risk at younger ages, reflecting differences in social support, economic security, pregnancy intention, and health status. For example, a meta-analysis reported an association between increasing age and PPD risk [42]. Other studies have reported similar findings. For example, women aged 32 years and older had a slightly increased risk of psychological distress during pregnancy and throughout the first 18 months of motherhood compared with women aged 25–31 years [43]. Similarly, Silverman et al. reported that the risk of depression increased with advanced maternal age at 1 year postpartum [8]. Muraca et al. also found that the prevalence of depression among recently delivered women was significantly higher in those aged 40–44 years than in those aged 30–35 years [44].

In our study, age was modeled continuously and may not capture non-linear patterns; additionally, given limited precision, small-to-moderate age effects may not have been detectable. Overall, our findings suggest that age alone is not a strong standalone predictor of current depressive symptoms in this sample once education is considered. While increasing age may raise vulnerability through the cumulative burden of childbearing and caregiving, older mothers may also benefit from greater parenting experience and coping skills. These opposing effects may explain why maternal age was not a strong independent predictor of current depressive symptoms in our sample.

Taken together, these results reinforce that commonly cited demographic correlates (income, parity, and age) may not show consistent independent associations with maternal depressive symptoms once more stable markers of disadvantage, particularly education, are accounted for.

### Public health implications

Practical implications of our study include integrating brief depression screening into routine maternal health encounters and prioritizing supportive referral pathways for mothers with limited formal education. Interventions that extend beyond the individual, such as family-informed counseling, community support mechanisms, and efforts to reduce stigma and improve mental health literacy, may be especially relevant. Evidence syntheses in LMICs underscore both the high burden of maternal depression and the urgency of scalable, context-appropriate support strategies [19,37].

### Future research

Future research should use probability-based sampling and longitudinal designs that repeatedly measure depressive symptoms across reproductive transitions and early childrearing, while also capturing relationship dynamics, social support quality, and gendered household roles. Such designs are better suited to disentangling temporality, reducing selection bias, and determining whether birth spacing exerts an independent effect on maternal mental health once structural determinants are adequately measured.

### Selection bias and generalizability

A major implication of our recruitment approach is that selection processes could affect the measured prevalence of both exposure and outcome, as well as their association. Recruitment occurred in selected areas during standard working hours. Mothers who are not present in these settings, due to caregiving responsibilities, limited mobility, employment schedules, cultural norms, or financial barriers, may be under-represented. This matters for both sides of the association. For example, women with very short spacing may be more home-bound due to childcare burden, potentially leading to under-representation of the most exposed group. Likewise, mothers experiencing depressive symptoms may be less likely to be in public settings or to engage with recruiters, which could underestimate depression prevalence. If these mechanisms operate concurrently, they could bias associations toward the null. As a result, findings should be interpreted as most applicable to mothers accessible in these recruitment settings, and prevalence estimates should not be treated as representative of all mothers in Karachi.

### Other limitations

This study has several limitations. First, its cross-sectional design precludes causal inference and clear temporal ordering; depressive symptoms may have predated or influenced birth spacing decisions (e.g., via pregnancy planning or contraceptive use), so reverse causation cannot be excluded. Second, measurement and reporting biases are possible: spacing was derived from participant-reported birth dates and may be misclassified due to recall error, and PHQ-9 symptoms were self-reported without clinical confirmation, with stigma and limited mental health literacy potentially leading to underreporting and bias. Third, although the study met its planned sample size, precision was limited (n = 187) with wide confidence intervals, and larger samples would be needed to detect smaller associations and support more complex models without overfitting. Fourth, we focused on spacing between the two most recent births and did not evaluate alternative metrics (e.g., the shortest inter-birth interval). Finally, refusal rates were not systematically recorded, and residual confounding remains possible despite adjustment for prespecified sociodemographic covariates.

## Conclusion

In this cross-sectional study of mothers in Karachi, shorter birth spacing (<24 months and 24–32 months) was not significantly associated with higher odds of probable depression (PHQ-9 ≥ 10) compared with spacing ≥33 months. In contrast, lack of formal education showed a consistent and statistically significant association with greater depressive symptom burden across multiple outcome definitions. Given the study’s design, convenience sampling, and limited precision, these findings should be interpreted as associations rather than causal effects. Larger, probability-based and longitudinal studies are needed to better quantify the relationship between birth spacing and maternal mental health and to inform targeted screening and support for higher-risk mothers.

## Abbreviations

CS: child spacing
WHO: World Health Organization
PPD: postpartum depression
PHQ-9: Patient Health Questionnaire-9
cOR: crude odds ratio
aOR: adjusted odds ratio
aPOR: adjusted proportional odds ratio
SES: socioeconomic status
